# Tou Nong San Attenuates Inflammation in TNBS-IBD Model by Inhibiting NF-*κ*B Signaling Pathway

**DOI:** 10.1155/2018/6929307

**Published:** 2018-06-25

**Authors:** Zhipeng Hu, Maoyi Yang, Qiaobo Ye, Kaihua Qin, Mingquan Wu, Rui Gu, Yingguang Zhou

**Affiliations:** ^1^Chengdu University of Traditional Chinese Medicine, Chengdu 610075, China; ^2^Basic Medical College, Chengdu University of Traditional Chinese Medicine, Chengdu 611137, China; ^3^Health Preservation and Rehabilitation College, Chengdu University of Traditional Chinese Medicine, Chengdu 611137, China; ^4^Sichuan Provincial Orthopedic Hospital, Chengdu 610041, China; ^5^Ethnic Medical College, Chengdu University of Traditional Chinese Medicine, Chengdu 611137, China

## Abstract

The incidence of inflammatory bowel disease (IBD), which predominantly comprises Crohn's disease and ulcerative colitis, is increasing worldwide. However, the treatment of IBD still faces great challenges. The involved NF-*κ*B is the main signaling pathway in human IBD and thus is a prime target. There is abundant evidence that Tou Nong San (TNS), which is a traditional Chinese medicinal decoction used for treating sores and carbuncles, has a positive effect on the inflammation. This study investigated the effects of oral administration of TNS on colitis induced by 2,4,6-trinitrobenzenesulfonic acid (TNBS) and the underlying mechanism(s). Quality control of the major compounds in TNS was performed by high-performance liquid chromatography, and six chemical constituents were identified in aqueous extracts. TNS led to improvements in weight loss and water and food intake in rats. The macroscopic and microscopic scores of rat tissues greatly decreased. Protein and mRNA levels of proinflammatory cytokines, including interleukin-17 (IL-17), tumour necrosis factor-*α*, IL-1*β*, and IL6, involved in the NF-*κ*B signaling pathway were greatly reduced. The results suggest that the anti-inflammatory effect of TNS is associated with the regulation of the NF-*κ*B signaling pathway, which contributes to the network pharmacological effect of TNS on human IBD in clinical practice.

## 1. Introduction

Crohn disease (CD) is a chronic inflammatory bowel disease (IBD). It is characterized by symptoms such as abdominal pain, frequently relapsing bleeding, diarrhea, and weight loss. The incidences of CD in both adults and children continue to climb, especially in recent decades [1]. The etiology of IBD is complex and multifactorial, but researches so far suggest that the interaction of genetic and other excluding genetic factors is associated with the onset of Crohn disease [2].

As with ulcerative colitis (UC), another type of IBD, the inflammation in colon is entailed in the pathogenesis of CD. According to current knowledge, the CD is a Th1/Th17 cytokine-mediated disorder [3]. The aberrantly activated T cells secrete specific cytokines. NF-*κ*B plays a prominent role in the induction of proinflammatory cytokines and inflammatory responses in CD [4]. It consists of homo- or heterodimers p50/p65 and an inhibitory I*κ*B protein. When the I*κ*B kinase complex (IKK*β*) is activated, I*κ*B is then phosphorylated and eventually degraded. The degradation of I*κ*B further leads to the activation of p50/p65. The p50/p65 then enters the nucleus and activates transcription of its downstream target genes including TNF-*α*, IL-1*β*, IL-6, IL-17, and other inflammatory mediators [5].

Based on the above understanding abstract, inhibiting inflammation is the major treatment strategy of CD. However, all the current medical therapies have their own limitations. Aminosalicylates are suitable only for modest patients [6, 7]; glucocorticoids have many side effects and do not provide patients with maintenance therapy [8]. Biological agents, though effective, can lead to serious infection [9]. As a classical and effective therapeutic means, Traditional Chinese Medicine (TCM) has its diversification and uniqueness, and TNS is the typical representation utilized in treating CD.

Tou Nong San (TNS) is a TCM formula, which is widely used in the clinical surgery in China to treat skin ulcer which refers to the treatment strategy of promoting eruption and draining toxin in peripheral body. Correspondingly, TNS has good effect in CD and it contains 5 Chinese herbal medicines, namely,* Radix astragali* 12 g,* Angelica sinensis* 6 g,* Ligusticum* 9 g, Spina Gleditsiae 4.5 g, and pangolin scales 3 g. In the past, pharmacological studies confirmed that the principal ingredients of TNS possess anti-inflammatory functional result in controlling diseases [10–20]. The pharmacologic mechanism underlying practical clinical application of TNS in CD remains unknown. Thus, our research is conducted to explore the anti-inflammatory property of TNS and the latent mechanism(s).

## 2. Materials and Methods

### 2.1. Experimental Design

Male SPF/SD rats (6-8 weeks old) were obtained from Chengdu Dashuo Co. (Chengdu, Sichuan, China) and given unconditional access to water and food before and during the study. Rats were weighed before the experiment and only those weighing between 200 and 210 g were qualified. Experiments were performed on the morning of the eighth day after the animals' arrival. To establish colitis, solution mixed with 100 mg/kg TNBS solution and isometric 50% ethanol was injected slowly into the colon of a rat via an 8 cm thin catheter [21]. And the rat was held in a head-down posture for 5 min after administration. In this way, the TNBS-ethanol enema was fully absorbed by the intestinal tract of rats. If an animal quickly (less than 5 min) excluded the solution, it would be removed from the rest of the study. About 12 hours after the administration of TNS, most of the rats developed clinical symptoms. If one rat did not develop colitis, it was removed from the experiment. Then the rats with colitis were randomly divided into four groups. Model group received normal saline (NS), and the other three experiment groups received 3.3 ml/kg, 6.6 ml/kg, and 13.2 ml/kg TNS decoction, respectively. All the solution was administrated orally. In order to ensure the reliability of the results, all the rats received everyday treatment and were sacrificed in the last day at the same time. All protocols of our experiment were approved by the Chengdu University of Traditional Chinese Medicine Experimental Animal Ethics Committee. The ethical committee number is 2017-06.

### 2.2. Macroscopic Assessment and Histological Analysis of Colitis

With their daily weight and water/food consumption recorded, the animals were sacrificed on the seventh day. The colon was quickly removed after sacrifice. The stool was opened longitudinally and cleared gently before a macroscopic assessment was given. Then, the samples of colonic tissue were divided into two parts.

The first one, about 0.5 cm long, was fixed in 4% paraformaldehyde and embedded in paraffin for hematoxylin-eosin (HE) and the second was stored at liquid nitrogen until use. Macroscopic assessment was performed according to the criteria of Minaiyan* et al*. (2014) and Vochyanova Z (2017) with some modifications as follows: (0) no inflammation; (1) only hyperemia; (2) edema without ulceration; (3) only one site of ulceration; (4) more than one site of ulceration; (5) severe ulceration > 2 cm. Microscopic assessment criteria are as follows: the severity of inflammation was graded as 0-3 for the extent of inflammation (mucosa, submucosa, and transmural layers) and 0-4 for crypt damage. Then the three parts would be added up to make a total score [22, 23].

### 2.3. Reagents

H&E staining solution was purchased from Nanjing Jiancheng Bioengineering Institute. The Rat IL-17 ELISA Kit, TNF-*α* ELISA Kit, IL-6 ELISA Kit, and IL-1*β* ELISA kit were purchased from MultiSciences (Lianke) Biotech Co. Ltd. (Hangzhou, China). The I*κ*B*α* (44D4) Rabbit mAb, NF-*κ*B p65 (D14E12) Rabbit mAb, and Phospho-IKK*α*/*β* (ser176/180) (16A6) Rabbit mAb were purchased from Cell Signaling Technology, Inc. (USA). The 5% TNBS solution was purchased from Sigma Co. (USA). Reference compounds including Campanulin, Isoflavone, Anthocyanins, Miscanthus, ferulic acid, astragaloside A, quercetin, coniferyl ferulate, Senkyunolide I, Senkyunolide A, z-ligustilide were purchased from Chengdu Pusi Biological Polytron Technologies Inc. (Chengdu, China). The purity of all the reference compounds was identified after being purchases.

### 2.4. Preparation of TNS

The composition of formula is shown in Table 1. Pangolin scales had not been added in our experiment because of their ethical controversy. 315 grams of TNS herbal slice was obtained from Chengdu Ji'ankang Medical Co. Ltd., Chengdu, China (the proportion of components in TNS formula was maintained). Herbal components of TNS were identified as* Radix astragali*,* Angelica sinensis*,* Ligusticum*, and Spina Gleditsiae. The medicine was soaked with 1260 ml of water and then the medicinal herbs would be decocted on the slow fire until it was condensed to about 157.5 ml. This process was repeated two times and the solutions were mixed to make TNS solution (about 315 ml), which would be stored for later use.

### 2.5. HPLC

The HPLC analysis was performed by validated methods based on linearity, limits of detection, quantification, reproducibility, and recovery. Chemical references were purchased from Chengdu Pusi Biological Polytron Technologies Inc. (Chengdu, China). With 98% of purity, these assays were confirmed to be accurate, reproducible, and sensitive.

In brief, samples were separated on a reverse-phase analytical column (Zorbax XDB-C8, 4.6 × 150 mm, 5 mm; Agilent Technologies). The mobile phase was acetonitrile and 0.1% aqueous acetic acid, and the flow rate was 0.1 mL/min. Chemical profiles and the water extract were analyzed by HPLC.

### 2.6. ELISA

The blood samples were centrifuged at a rate of 3500 r/rain for 10 min at 4°C temperature and supernatant was obtained and stored at −80°C until being assayed. Levels of IL-1*β*, TNF-*α*, IL-6, and IL-17 were measured with enzyme-linked immunosorbent assay (ELISA) kits according to the manufacturer's protocols.

### 2.7. RT-qPCR

A specimen of inflammatory tissue in colon was cut and total RNA was obtained according to the TRIzol reagent manufacturer's instructions. Reverse transcription was carried out for 1 h at a temperature of 37°C in a reaction mixture containing 2.0 *μ*g total RNA, 10 mM dNTPs, 0.5 *μ*g oligo (dT) primer, 200 units M-MLV reverse transcriptase, and 25 units of RNase inhibitor. RT-qPCR was performed with the SYBR qPCR kit. The mRNA levels of IL-17, TNF-*α*, IL-1*β*, and IL-6 were normalized to *β*-actin. The following primers were used for the reverse transcriptase-PCR analysis: *β*-actin: forward primer, 5′-TGGAATCCTGTGGCATCCATGAAAC-3′, and reverse primer, 5′-TAAAACGCAGCTCAGTAACAGTCCG-3′; TNF-*α*: forward primer, 5′-GGCAGGTCTACTTTGGAGTCATTGC-3′, and reverse primer, 5′-ACATTCGAGGCTCCAGTGAATTCGG-3′; IL-17: forward primer, 5′-ATCAGGACGCGCAAACATG-3′, and reverse primer, 5′-TGATCGCTGCTGCCTTCAC-3′; IL-6: forward primer, 5′-TCCAGTTGCCTTCTTGGGAC-3′, and reverse primer, 5′-GTACTCCAGAAGACCAGAGG-3′; IL-1*β*: forward primer, 5′-AGCCCATCCTCTGTGACTCATG-3′, and reverse primer, 5′-GCTGATGTACCAGTTGGGGAAC -3′.

### 2.8. Western Blot Analysis

To evaluate p-IKK*β*, I*κ*B, p65, and AP-1 protein level, 0.2 g of tissue was removed from colon and was washed with precooled PBS for three times. The tissue was ground into small pieces and was stirred in 10 volumes of lysis buffer and centrifuged at 4°C for 10 mins. The total protein was then isolated with electrophoresis with sodium dodecyl sulfate-polyacrylamide gel (SDS-PAGE), and the nucleus protein was isolated by a specific kit. After being separated, the proteins were transferred onto polyvinylidene difluoride (PVDF) membranes and blocked with 5% nonfat dry milk in TBST (20 mM Tris-HCl, 150 mM NaCl, and 0.05% Tween-20) for 1 hour at room temperature. Then the membranes were incubated with primary antibodies overnight at 4°C and with HRP conjugated goat anti-rabbit secondary antibodies for 2 h at room temperature on the second day. The membranes were washed for three times with TBST for 10 mins before the p-IKK*β*, I*Κ*B, p65, and AP-1 antibodies were used to detect the respective proteins.

### 2.9. Statistical Analysis

SPSS 18.0 software was used to analyze all the results of the research. Data are expressed as means ± SD. Kruskal-Wallis H test was conducted to evaluate the scores of macroscopic damage and histological score. For the rest of the results, one-way ANOVA test followed by the post hoc Dunnett's multiple comparisons test was used to analyze statistical difference between groups. The difference was considered statistically significant if* P *< 0.05.

## 3. Results

### 3.1. Quality Control of the TNS by HPLC

**Figure 1:**
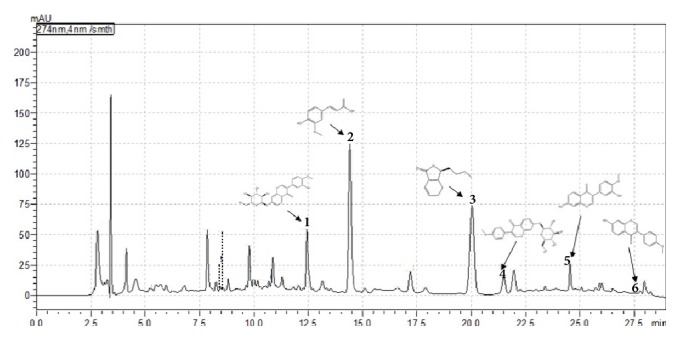
The 6 major chemical constituents in TNS decoction. From left to right successively were calycosin-7-glucoside (1), ferulic acid (2), Senkyunolide I (3), ononin (4), calycosin (5), and ferromagnetic (6).

**Figure 2:**
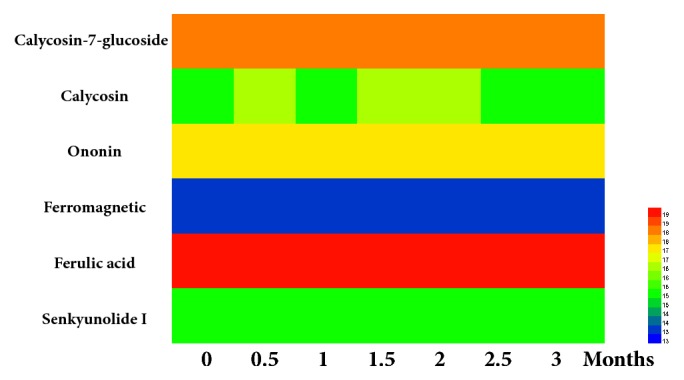
The stability of 6 major chemical constituents in quality control towards TNS decoction at 6 time points.

Since TNS had complex composition and was processed by the traditional method of TCM, the main bioactive compounds of TNS in water extracts were identified and determined by HPLC. The transfer rates of six compounds, calycosin-7-glucoside (1), ferulic acid (2), Senkyunolide I (3), ononin (4), calycosin (5), and ferromagnetic (6), were, respectively, 46.2%, 40.6%, 71.6%, 39.2%, 79.3%, and 20% (Figure 1). In addition, the stability of the six compounds was also determined and evaluated at the 0, 0.5, 1, 1.5, 2, 2.5, and 3 months that accompanied the complete test. Stability results showed that main active compounds in research sample were balanced and the RSD was 0.49%, 0.22%, 0.59%, 0.29%, 0.29%, and 2.17% (Figure 2). The study was the primary foundation and guaranteed the reliability of the whole work.

### 3.2. TNS Inhibits Clinical Manifestation of TNBS-Induced Colitis

**Figure 3:**
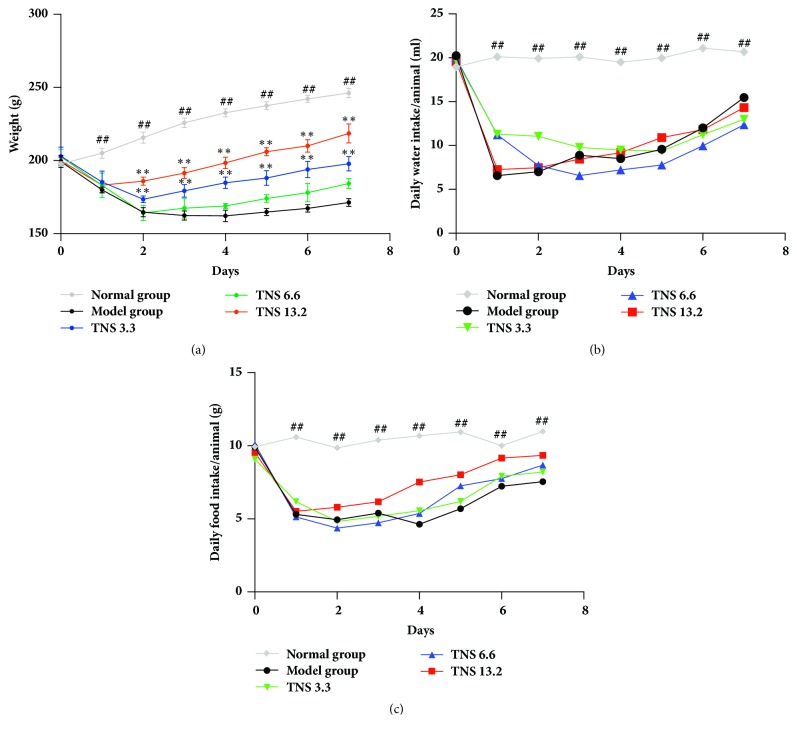
Effect of different doses of TNS on clinical manifestation of TNBS-induced rats. (a) Everyday weight loss of five groups. (b) Everyday water intake of five groups. (c) Everyday food intake of five group. Values are presented as means ± SD (*n* = 10). Model group versus normal group: ^#^*P* < 0.05; ^##^*P* < 0.001; TNBS versus treated groups: *∗* represents* P* < 0.05; *∗∗* represents* P* < 0.01.

After the enema of TNBS, most of the rats developed colitis with the clinical manifestations including diarrhea and weight loss. The first sign of colitis appeared at about the 12th hour after the administration and the peak of clinical manifestation appeared on the second day. TNS decoration was administrated to the rats after the sign of colitis appeared. In order to ensure the reliability of the results, all the treatments were conducted at the same time. Like in the model group, the peak of clinical manifestation in the treatment group occurred on the second day, but the recovery of the treatment group was significantly enhanced. We recorded the weight loss and everyday food and water intake of rats. The weight loss of the treated groups reduced, as is shown in Figure 3(a), compared with the model group. There is no significant difference about water (Figure 3(b)) and food intake (Figure 3(c)) between the model group and the treated groups (*P* > 0.05).

### 3.3. TNS Represses Tissue Damage of TNBS-Induced Colitis

**Figure 4:**
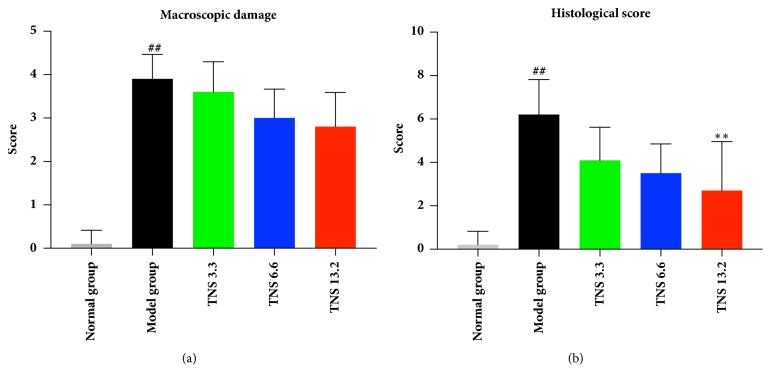
TNS reduced tissue damage of TNBS-induced colitis. (a) Macroscopic score of rat after 7 days' use of TNS. (b) Histological assessment. The criteria of assessment are described in Materials and Methods. Values are presented as means ± SD (*n* = 10). Model group versus normal group: ^#^*P *< 0.05; ^##^*P *< 0.001; TNBS versus treated groups: *∗* represents* P *< 0.05; *∗∗* represents* P *< 0.01.

**Figure 5:**
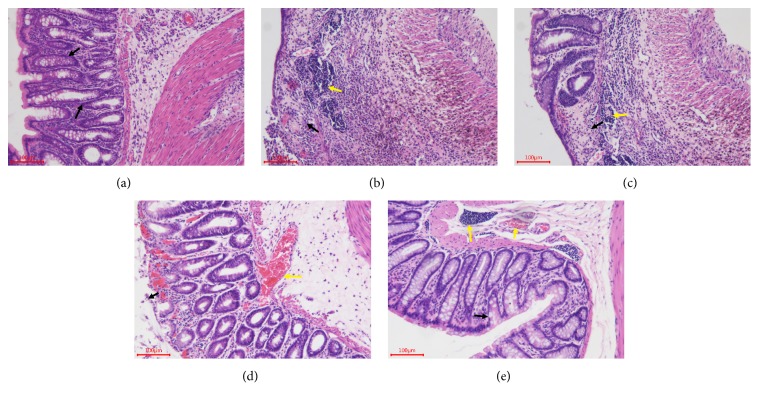
Histological results of colon tissue. (a) Normal group, (b) model group, (c) TNS (3.3 ml/kg), (d) TNS (6.6 ml/kg), and (e) TNS (13.2 ml/kg). In normal group, the epithelium of colon was integrated and there was no inflammation cell. The black arrows refer to the epithelium of colon. In model group, the epithelium was damaged completely and there were a lot of inflammatory cells. In TNS group, as the dose of TNS increases, the gut barrier was gradually repaired. The extent of inflammation in the mucosa and submucosa was reduced as the dose of TNS increased, as shown by the yellow arrows.

The above data demonstrated a clinical improvement after the use of TNS. Then the macroscopic score of each rat was calculated. There was no statistical difference between TNS group and model group. Then the intestinal tissue was observed by optical microscopy and H&E staining techniques. According to the histological analysis, the model group was successfully induced into colitis (*P* < 0.0001) (Figure 4(b)). The epithelial cells were necrotic, resulting in a disrupted colonic architecture and ulcers in the epithelium (Figure 5(b)) compared to the normal group (Figure 5(a)). TNS helps to repair the epithelium of colon, especially in the high-dose group (Figures 5(c), 5(d) and 5(e)). In the model group, the mucosa and submucosa are marked by inflammatory cell infiltration (Figure 5(b)). After the use of TNS, the inflammatory cells were greatly reduced (Figures 5(c), 5(d) and 5(e)). The scores in high-dose group decreased significantly compared to the model group (decreased by 56.4%,* P* = 0.0038) (Table 2).

### 3.4. TNS Reduced Proinflammatory Cytokines in TNBS-Induced Colitis

**Figure 6:**
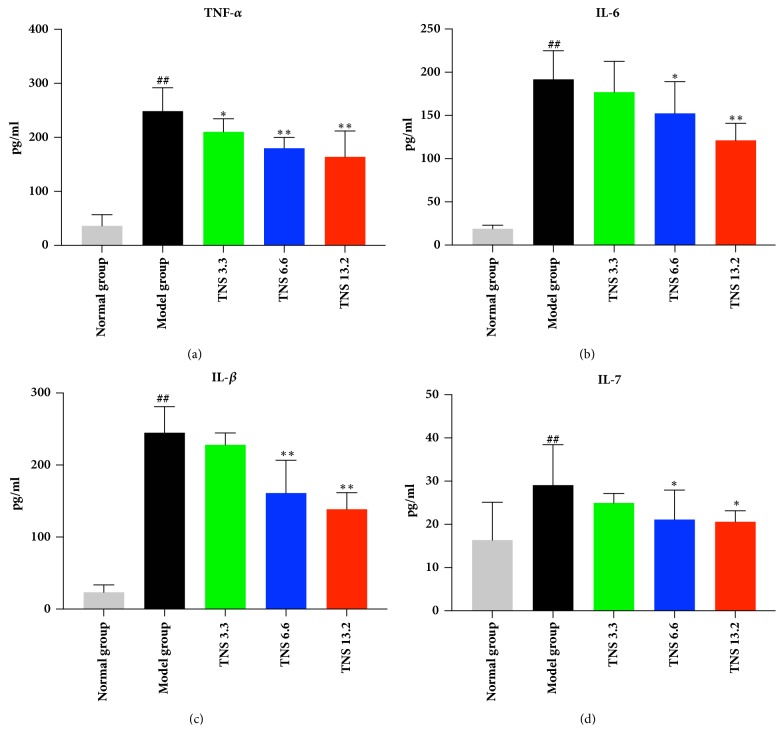
Effect of TNS on serum inflammatory cytokines including (a) TNF-*α*, (b) IL-6, (c) IL-1*β*, and (d) IL-17. The level of these cytokines in supernatant was assayed by ELISA. Values are presented as means ± SD (*n* = 10). Model group versus normal group: ^#^*P* < 0.05; ^##^*P* < 0.001; TNBS versus treated groups: *∗* represents* P* < 0.05; *∗∗* represents* P *< 0.01.

**Figure 7:**
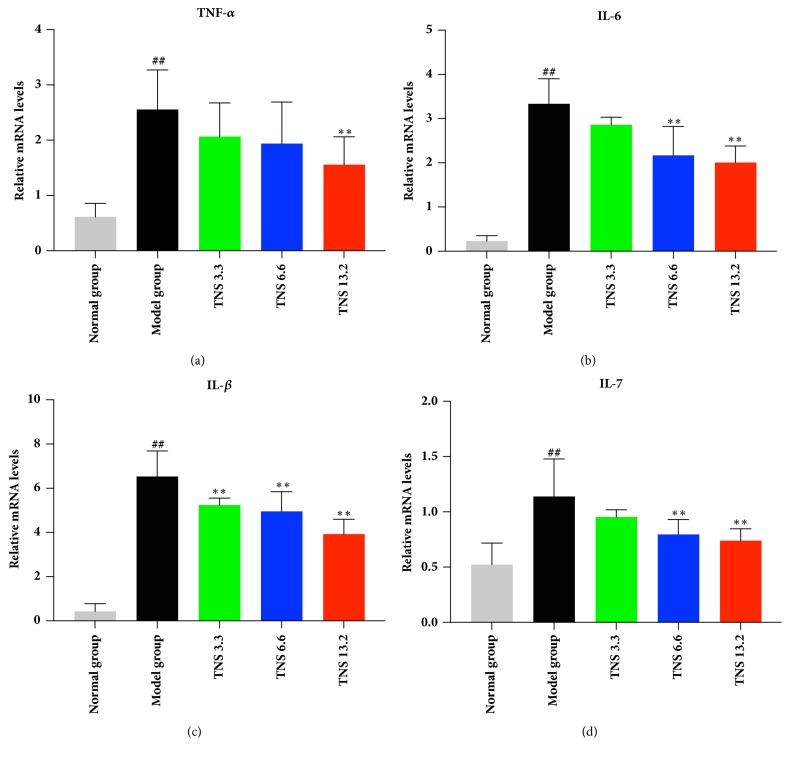
Effect of TNS on colon cytokines' mRNA levels in TNBS-induced colitis including (a) TNF-*α*, (b) IL-6, (c) IL-1*β*, and (d) IL-17. Colon tissue was removed from rats after rats were sacrificed. The processing methods of sample were described in Materials and Methods. The mRNA levels were determined by RT-qPCR analysis. Values are presented as means ± SD (*n* = 10). Model group versus normal group: ^#^*P* < 0.05; ^##^*P* < 0.001; TNBS versus treated groups: *∗* represents* P* < 0.05; *∗∗* represents* P* < 0.01.

Since the inflammation was reduced by TNS, our investigation moved on to the level of proinflammatory cytokines. Crohn disease is characterized by a Th-1- and Th-17-dominated immune response. Therefore, the following proinflammatory cytokines in serum were investigated by ELISA: TNF-*α*, IL-6, IL-1*β*, and IL-17. The results are shown in Figures 6 and 7. The serum concentration of these cytokines was downregulated by TNS. TNF-*α* in the medium- and the high-dose group decreased sharply by 28% and 34%, respectively (*P* < 0.0001 in these two groups) (Figure 6(a)), and the levels of IL-1*β* in the medium- and the high-dose group decreased by 28.5% (*P *<0.0001) and 39.3% (*P *< 0.0001) (Figure 6(c)). The levels of IL-6 in the three treated groups, compared with the model group, were downregulated by 7.8% (*P* = 0.6057), 20.6% (*P* = 0.0129), and 36.7% (*P* < 0.0001), respectively (Figure 6(b)). The mRNA expression of these cytokines in the colon was analyzed by RT-qPCR. The concentration of TNF-*α* mRNA in high-dose group decreased by 38.6% (*P* = 0.0019) (Figure 7(a)). The mRNA level of IL-6 in medium- and high-dose group decreased by 35% (*P* < 0.001) and 39.8% (*P* < 0.001). The IL-1*β* mRNA in all the three treatment groups decreased significantly (*P* values are all less than 0.001) (Figures 7(b) and 7(c)). The serum and mRNA concentrations of IL-17 were lower than the other three cytokines, and they were significantly reduced, especially in the high-dose group (*P* = 0.0235 and* P* < 0.001, resp.) (Figures 6(d) and 7(d)).

### 3.5. TNS Inhibits the NF-*κ*B Signaling Pathway

**Figure 8:**
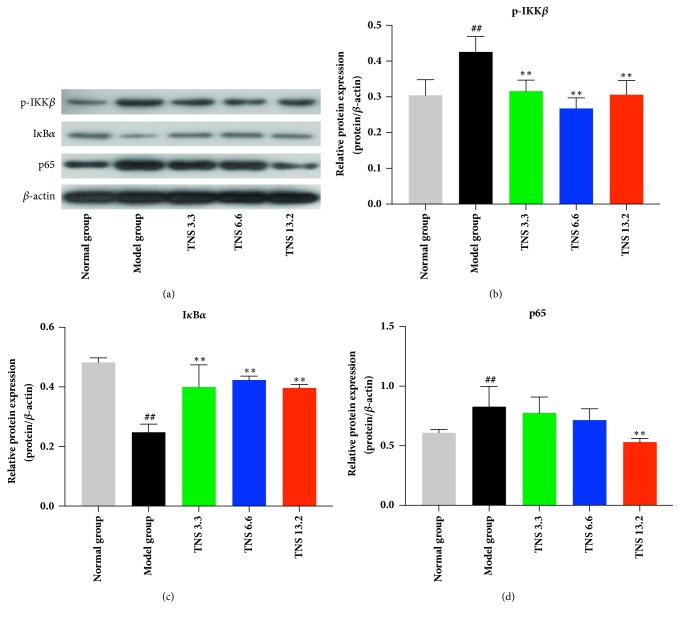
Effect of TNS on expression of p-IKK*β*, IKK*α*, and p65 protein in TNBS-induced colitis. Colon tissue was removed from rats after rats were sacrificed. The processing methods of sample were described in Materials and Methods. The protein levels were determined by western blot analysis. Values are presented as means ± SD (*n* = 10 ). (a) Western blot analysis of p-IKK*β*, IKK*α*, and p65. (b) Relative protein expression of p-IKK*β* compared to *β*-actin. (c) Relative protein expression of IKK*α* compared to *β*-actin. (d) Relative protein expression of p65 compared to *β*-actin. Model group versus normal group: ^#^*P* < 0.05; ^##^*P* < 0.001; TNBS versus treated groups: *∗* represents* P* < 0.05; *∗∗* represents* P* < 0.01.

Based on the results that TNS inhibits the serum concentration and mRNA levels of proinflammatory cytokines, attempt in furthering the exploration into signaling pathway involved in the regulation of TNS was made. The nuclear protein of colon tissue was extracted and western blot analysis was conducted to investigate the level of p65 and AP-1, the two major transcription factors in inflammation response. The protein level of p65 in model group was higher than that of the control group (26%,* P* < 0.0001) and the treatment group and especially was higher than that of the dose group (35.9%,* P* < 0.0001) (Figures 8(a) and 8(d)). The level of cytoplasmic protein p-IKK*β* decreased by 26% in low-dose group (*P* < 0.0001), 37% in medium-dose group (*P* < 0.0001), and 28% in high-dose group (*P* < 0.0001) (Figures 8(a) and 8(b)). The level of cytoplasmic protein I*κ*B*α* increased by 60.7% in the low-dose group* P *< 0.0001), 70.2% in the medium-dose group (*P *< 0.0001), and 60% in the high-dose group (*P < *0.0001) (Figures 8(a) and 8(c)). The nuclear concentration of AP-1 in both the model and the treatment groups was at a low level (data not shown).

## 4. Discussion

This study was conducted to investigate the anti-inflammatory property of TNS and its underlying mechanism(s). This experiment explored whether the oral administration of TNS could decrease inflammation in colon induced by TNBS. Previous TNS-related researches have established a basis from the clinic application of TNS in CD, which promoted the further investigation. The TNBS-induced colitis model shows similarities to CD in clinical manifestations and histological findings and has been utilized as an ideal model to explore the pathogenesis, pathology characteristics, and curative effect of IBD [21]. Before starting the experiment, the quality control of TNS based on identifying the components in decoction was conducted by HPLC. Water extraction is still commonly used in clinical practice, so the compound of TNS water extraction was investigated. The major compounds of TNS are as follows: calycosin-7-glucoside, calycosin, ononin, formononetin, ferulic acid, and Senkyunolide I. Advances in pharmacological research about TNS revealed that the above bioactive components possess a good effect on inflammation. The efficacy of these components in TNS decoction plays a main role that leads to its efficacy. Certainly, these compounds could not fully reflect the overall perspective of TNS in treating CD.

As illustrated data in experiment, the clinical condition of rats was improved after the use of TNS. The weight loss and food/water intake indicate that short-term use of TNS cannot improve appetite and weight of colitis rats. To investigate its visual efficacy from histological perspective, the macroscopic and microscopic scores were obtained—TNS has significant intervention effect and could reduce tissue damage.

Inflammation is a major inducing factor in the pathogenesis of IBD and causes intestinal tissue injury in IBD. As important inflammatory biomarkers, related cytokines including TNF-*α*, IL-1*β*, IL-6, and IL-17 play an important role in inflammatory response. In previous data, it can be seen that the tissue damage of rats in experiments group was milder than that of the model group and so was the lymphocyte infiltration reflected by the H&E staining. So, the levels of cytokines in serum were detected by ELISA and in colon by PCR. Compared with the model group, the TNF-*α*, IL-1*β*, and IL-6 in experiments group decreased significantly, especially the high-dose group.

The results showed clearly that TNS downregulated the major inflammatory cytokines in both protein and mRNA levels, thus confirming a good anti-inflammatory effect in IBD. However, the IL-17 levels in all groups were low. This was in line with previous studies, which demonstrates that the single-dose-TNBS model mainly resulted in an acute local inflammatory response characterized by Th1 cytokines secretion [24, 25].

**Figure 9:**
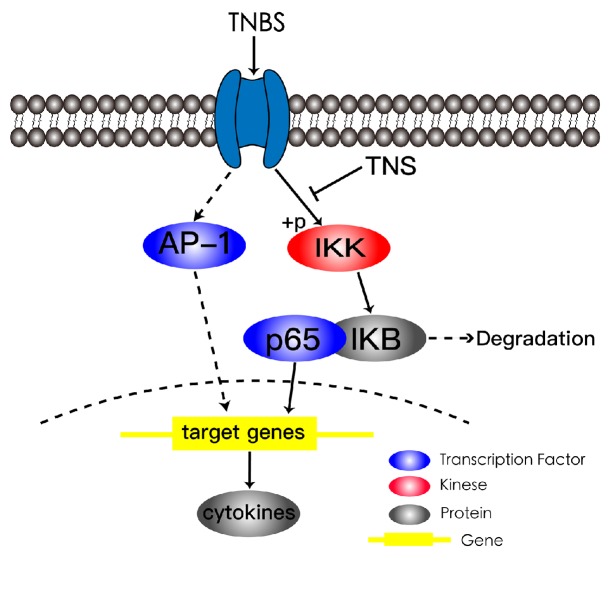
Schematic diagram depicts the possible mechanism for the anti-inflammatory effect of TNS. There are two transcription factors that regulate the transcription of inflammatory cytokines: AP-1 and NF-*κ*B. TNS inhibits the phosphorylation of IKK and thus inhibits the degradation of I*κ*B. Then the activation and translocation of p65 are inhibited by I*κ*B. Eventually the transcription of target genes is reduced.

NF-*κ*B is an important signaling pathway for the transcription of inflammatory cytokines in IBD. At baseline, the p60/p65 protein bound with its inhibitor, I*κ*B*α*. The protein complex was located in cytoplasm in an inactivated state. When the receptor in the cell membrane is activated by triggering factor, the I*κ*B kinase (IKK) complexity is phosphorylated, resulting in the degradation of I*κ*B*α*. The p60/p65 is then set free and enters the nucleus to activate target gene. To investigate whether TNS inhibited inflammation by inhibiting NF-*κ*B signaling pathway, the level of p65 in nucleus was analyzed. After being treated with TNS, p65 in nucleus decreased significantly, which suggested that TNS could suppress inflammation by inhibiting the activation or translocation of p65. Then the levels of p-IKK*β* and I*κ*B*α* were detected by immunoblot analysis. The increased degradation of I*κ*B*α* and phosphorylation of IKK*β* implied that TNS inhibited p65 mainly by inhibiting the phosphorylation of IKK*β* and thus the degradation of I*κ*B*α* instead of inhibiting the translocation of p65 (Figure 9).

In addition to NF-*κ*B, there is another inflammatory transcription factor that is involved in the mediation of proinflammatory cytokines, activator protein-1 (AP-1). To get rid of the possibility that the anti-inflammatory effect of TNS is due to AP-1, we detected AP-1 level at the same time. The AP-1 level was not activated in both the model and the treatment groups, indicating that AP-1 is not involved during the entire experiment and TNS attenuates inflammation mainly by diminishing NF-*κ*B signaling pathway (Figure 9).

There are related pharmacological studies on the components of TNS in inflammatory model. In these studies, Senkyunolide, calycosin, and ononin showed anti-inflammatory effects including inhibiting TNF-*α*, IL-1*β*, and IL-6 and in turn other downstream cytokines by targeting NF-*κ*B and other signaling pathways like cJNK and MAPK. These researches confirm the anti-inflammatory effect of TNS and indicate that the therapeutic effects of TNS have similar mechanism. Their anti-inflammatory property plays a role in many disease models including stroke-induced neuroinflammation model, DSS-induced colitis model, and lipopolysaccharide-stimulated macrophages model.* Chuān xiōng* (Rhizoma Chuanxiong) capsule that contains Senkyunolide could decrease inflammation in atherosclerotic rats through PI3K/Akt and NF-*κ*B pathway.* Dāng Guī Bŭ Xuè Tāng *(Chinese Angelica Blood-Supplementing Decoction, composed of* huáng qí* and* dāng guī*) possesses a more extensive immune-regulatory effect [10–20].

Taken together, the anti-inflammatory effect and therapy manifestation of TNS were associated with the downregulation of the classical NF-*κ*B signaling pathway.

## 5. Conclusions

To summarize, the results reported here demonstrate for the first time that TNS attenuates inflammation in rat model of Crohn disease by targeting NF-*κ*B signaling pathway. Our research has the potential to give pharmacological support for the application of TNS in CD.

## Figures and Tables

**Table 1:** The composition of TNS.

Pharmaceutical	Pīn yīn	Composition
*Radix astragali*	*huáng qí*	32%
*Angelica sinensis*	*Dāng Guī*	24%
*Ligusticum*	*chuān xiōng*	24%
Spina Gleditsiae	*Zào Jiǎo Cì*	12%
Pangolin scales^*∗*^	*Chuān Shān Jiǎ*	8%

*∗*Pangolin scales had not been added in our experiment because of their ethical controversy.

**Table 2:** Macroscopic and histological scores. TNS reduced tissue damage of TNBS-induced colitis. The criteria of assessment are described in Materials and Methods. Values are presented as means ± SD (*n* = 10). Model group versus normal group: ^#^*P *< 0.05; ^##^*P *< 0.001; TNBS versus treated groups: *∗* represents *P *< 0.05; *∗∗*  represents *P* < 0.01.

	**Macroscopic damage** **(Mean ± SD)**	**Histological scores**			
		**Severity of inflammation and infiltration of immune cells ** **(Mean ± SD)**	**Inflammation extent** **(Mean ± SD)**	**Crypt damage ** **(Mean ± SD)**	**Total ** **(Mean ± SD)**
Normal group	0.1 ± 0.3	0.1 ± 0.3	0.1 ± 0.3	0 ± 0	0.2 ± 0.6

Model group	3.9 ± 0.54^##^	2.3 ± 0.78^##^	2.3 ± 0.78^##^	1.6 ± 0.49^##^	6.2 ± 1.53^##^

TNS 3.3	3.6 ± 0.66	1.4 ± 0.5	1.4 ± 0.49	1.3 ± 0.64	4.1 ± 1.58

TNS 6.6	3 ± 0.63	1.2 ± 0.4*∗*	1.2 ± 0.4*∗*	1.1 ± 0.54	3.5 ± 1.28

TNS 13.2	2.8 ± 0.79	0.9 ± 1.2*∗∗*	0.9 ± 0.7*∗∗*	0.9 ± 0.7	2.7 ± 2.1*∗∗*

## Data Availability

The data used to support the findings of this study are available from the corresponding author upon request.
